# Perspectives on expert generalist practice among Japanese family doctor educators: a qualitative study

**DOI:** 10.3399/BJGPO.2021.0011

**Published:** 2021-05-19

**Authors:** Makoto Kaneko, Ai Oishi, Noriaki Sawa, Greg Irving, Yasuki Fujinuma

**Affiliations:** 1 Lecturer, Department of Health Data Science, Yokohama City University, Kanagawa, Japan; 2 Department of Family and Community Medicine, Hamamatsu University School of Medicine, Shizuoka, Japan; 3 Family Doctor, Primary Palliative Care Research Group, Usher Institute of Population Health Sciences and Informatics, University of Edinburgh, Edinburgh, UK; 4 GP Partner, Riverside Medical Centre, Castleford, UK; 5 Senior Lecturer, General Practice, Health Research Institute, Edge Hill University, Ormskirk, UK; 6 Family Doctor, Centre for Family Medicine Development, Japanese Health and Welfare Co-operative Federation, Tokyo, Japan

**Keywords:** expert generalist practice, family medicine, general practice, specialty, family doctors, physicians, family, Japan

## Abstract

**Background:**

Expert generalist practice (EGP) is increasingly being viewed as the defining expertise of generalist care. In Japan, several prominent family doctors consider it important and relevant in the Japanese context. However, no study has examined Japanese family doctor educators’ perceptions of EGP.

**Aim:**

To explore Japanese family doctor educators’ perceptions of EGP.

**Design & setting:**

A qualitative study among family doctor educators in Japan.

**Method:**

Focus group interviews were conducted using a semi-structured interview guide following a short lecture on EGP. A qualitative description method was adopted and the framework method was used to conduct thematic analysis.

**Results:**

Participants were 18 family medicine doctor educators, including 11 directors and six associate directors of family medicine training programmes. The results suggested that the concept of EGP was important and applicable to primary care in Japan. Participants’ perceptions on EGP pertained to the following four areas: impact of EGP, triggers for EGP, enablers for EGP, and educational strategies for EGP.

**Conclusion:**

The concept of EGP may be useful in clinical practice in Japan, especially in complex patient care. A clearer framework for or description of EGP, and of non-traditional methods, such as ascetic practice and awareness of the self, were proposed as possible educational strategies.

## How this fits in

EGP describes expertise in generalist medical practice. In Japan, although some family doctors emphasise the importance of EGP, no study has examined the Japanese family doctor educators’ perspectives on EGP. This study revealed that the concept of EGP was important and applicable to primary care in Japan.

## Introduction

Specialists and medical students, as well as family doctors themselves, repeatedly ask the question, ‘What is the expertise of family doctors?’^1–4^ One answer is the concept of EGP, proposed by Reeve *et al* ^5^ in the UK, where family doctors make up the largest group of medical generalists. EGP is a form of practice that utilises the philosophy of medical generalism, and it primarily focuses on the principle of ‘person-centred decisionmaking’ and the practice of ‘interpretive medicine’.^5–7^ Whole-person, individually-tailored clinical decisionmaking is seen as the defining expertise of generalist care internationally.^8^ Such expertise should be fostered through formal training, not merely experiential learning.^5^

In Japan, like many other countries, generalist care is seen as increasingly important.^9^ However, its role and responsibilities are not well defined.^10^ Also, there is no gatekeeping system.^10^ No system of registration (patient lists) exists, and there is no formal generalist training, as primary care doctors are usually ex-hospital specialists who have subsequently opened up clinics in their specialty.^11^ There are voluntary training programmes and examination for family doctors, which is managed by the Japan Primary Care Association (JPCA), the Japanese professional body of family medicine.^10^ There are now more than 800 JPCA-certified family doctors.^12^ With this as a background — and as part of the response to the challenges of Japan’s rapidly ageing society, such as increasing multimorbidity — specialist training for generalist practice was introduced formally as part of a new board certification system for medical specialties in 2018.^10^ The provision of patient-centred care has become one of the core learning objectives of this generalist training.^13^ Influential Japanese family doctors have reported their views on the importance and relevance of EGP in the Japanese context, which has attracted much attention.^14,15^ However, no study has examined family doctor educators’ views on EGP in Japan.

Therefore, the present study aimed to qualitatively describe Japanese family doctor educators’ perceptions of EGP. It focused on understanding how Japanese family doctor educators perceive this concept and what educational strategies they propose to enable the development of EGP in Japan.

## Method

### Design

This study employed a qualitative design.

### Present research team and reflexivity

The four authors, MK (MD, PhD), AO (MD, MSc), NS (MBChB), and YF (MD), participated as facilitators in the focus group interviews. All four authors are practising family doctors; one is female. The authors had previous experience of conducting interviews for qualitative research. The authors were conscious that their positive views on EGP might influence the planning of this study and interpretation of the findings. Thus, perceptions towards EGP were repeatedly reflected on and discussed among the authors in the processes of planning and conducting the study. Although some authors knew some participants before the focus group interviews, the interviews were aimed to be conducted in an open and supportive atmosphere to allow all the participants to express their own opinion without the influence of the authors’ opinions.

### Data generation

An email invitation was sent to a mailing list of directors and associate directors of family medicine training programmes to recruit family doctor educators. All applicants participated in the study, and the authors obtained written informed consent from the participants.

One-time focus group interviews were conducted with only the authors and the participants during the Japan Primary Care Associations’ Annual Conference, which was held on 18 May 2019. During the conference, one of the authors delivered a 40-minute lecture on the introduction of the concept of EGP, as defined by Reeve *et al,* and its application to the Japanese setting to all participants before conducting the interviews. After the lecture, the participants and facilitators were divided into four groups of similar age. A semi-structured interview guide was prepared (Box 1). Al l interviews were audio-recorded, and field notes were made during and after the interviews.

Box 1 **Interview guide. EGP = expert generalist practice**

**Table:** 

What is the significance of EGP?Would EGP have any impact on primary care in Japan?Do you think you practise EGP? If so, in what situations?How would you teach EGP? Do you teach it?Are there any barriers to practising or teaching EGP? How do you overcome these barriers?What would you like to learn about EGP?What would you like to add to the EGP lecture we presented today?

### Data analysis

A qualitative description method was adopted to explain Japanese family doctors’ views on EGP.^16,17^ In addition, the framework method was used to conduct a thematic analysis.^18^ Following data familiarisation, two authors (MK and AO) held weekly meetings to review all transcripts and prepare an initial list of codes. The two authors then independently coded two focus group interviews each, and conducted meetings to resolve any disagreements and reach coherence.^19^ After all transcripts were coded, a framework matrix^18^ was created to examine the relationships among emerging themes. Quotes or questions identified during the analysis were recorded and used in the final interpretation of the data. It was considered that the data reached the point of coherence because similar themes repeatedly emerged from different groups.

NVivo (version 12) was used for the coding and analysis. Following the analysis, participants conducted a member check to verify the appropriateness of the interpretations.

## Results

The study recruited 18 participants, with a median age of 43 years (interquartile range [IQR] 37.5–48 years), and a median number of years of clinical experience of 17 years (IQR 12.5–22 years). All participants were family doctor educators in Japan, with 11 directors and six associate directors of family medicine training programmes. Ten participants practised mainly in clinics, and eight practised in hospitals. Participants were divided into four groups for the focus group interviews, which were conducted in Japanese, each lasting for approximately 40 minutes. Participants’ characteristics have been presented in Table 1. In the quotes presented in this article, the group participant ID has been added after the quotes to indicate the speaker (that is, ’A1’ indicates Group A, Participant 1).

**Table 1.** Participants’ characteristics

Group	Participant ID	Age, years	Sex	Clinical experience, years	Position	Certification	Number of alumni in the programme
A	1	36	F	10–14	Associate director	FM	10–19
A	2	38	M	10–14	Director	FM	0–9
A	3	35	M	10–14	Associate director	FM	10–19
A	4	39	M	10–14	Associate director	FM	0–9
A	5	35	M	10–14	Associate director	FM	≥50
B	6	53	M	≥20	Director	None	0–9
B	7	50	M	≥20	Director	None	10–19
B	8	37	M	10–14	Director	GIM,Rheumatology	0–9
B	9	43	M	15–19	Director	Home care medicine	0–9
C	10	48	M	≥20	Director	FM, GIM,Home care medicine	0–9
C	11	47	M	≥20	Director	GIM,Geriatrics	0–9
C	12	43	M	15–19	Director	FM, GIM	30–39
C	13	39	M	15–19	Associate director	FM	20–29
C	14	44	M	10–14	Associate director	FM, GIM	0–9
D	15	47	M	≥20	Director	None	0–9
D	16	44	M	15–19	Director	GIM, Diabetes	0–9
D	17	42	M	15–19	Director	Home care medicine	0–9
D	18	52	M	15–19	Attending	None	0–9

FM Family medicine GIM General internal medicine

### Impact of EGP

Overall, participants were able to understand and relate to the concept of EGP; however, they thought that not all family doctors would have a good understanding of EGP:

*‘Not all family medicine trainees will be interested in EGP* *.* *’* (D18)

*‘Not all family doctors will be able to fully understand the concept of EGP. Some might be able to* *.* *’* (D17)

They deemed EGP to be intuitively important. Some described it as an area to be pursued in the future. Participants expressed four positive impacts and one negative impact of EGP.

#### Positive impact

##### Verbalisation of expert’s practice

Participants felt that EGP enabled them to verbalise what they did in their daily practice:

*‘As I listened to the concept of EGP, I thought about my own practice, thinking “yes that’s right, that’s right* *.”* *’* (C13)

*‘There is a refreshing sense of clarity as our daily practice is verbalised by the concept of EGP* *.* *’* (B9)

They also felt that the verbalisation and conceptualisation of EGP made it easier for them to learn the nature of consultations:

*‘*[EGP is] *the verbalisation of experienced doctors* *.* *’* (B8)

They also recognised that EGP would be useful for professional development after the completion of family medicine training:

*‘Acquiring EGP can be a next step for those who completed their family medicine training.’* (B9)

They also felt that adopting EGP would allow them to teach junior doctors what they had not been able to teach before.

##### Effective use of resources

Participants mentioned that they had seen patients with difficult and complex issues who could be managed better with EGP skills:

*‘As we expect to see more patients with complex problems or multi* *morbidities in the future, it will be increasingly important for us to develop the skills and techniques to be able to deal with these patients effectively* *.* *’* (B7)

One participant noted that family doctors tended to utilise a similar approach for patients with simple and those with complex problems. This led young and less experienced family doctors to spend more time with patients with simple problems. Participants reported that EGP enabled them to distinguish patients with simple problems from those with more complex issues, who may benefit more from EGP:

*‘In terms of managing complex cases, EGP can help clinicians who are struggling with difficult problems, so I think it will have an impact in that sense* *.* *’* (C11)

In addition, they reported that EGP would allow them to verbalise and share ideas on managing patients with complex needs with other doctors. They mentioned the potential of EGP to maximise available resources:

*‘I feel that dealing with difficult, complex cases in areas with inadequate resources will help the creation of this kind of approach in my mind* *.* *’* (A5)

##### Care integration

Participants considered EGP to have a multifaceted impact on care integration in the community:

*‘I think it’s a win-win for patients, carers, and doctors* *.* *’* (C14)

In addition, EGP was considered a useful guide for care integration in hospitals and clinics:

*‘The concept of EGP needs to be understood and shared also by hospital doctors working at the point of contact with the community so that care will be more vertically integrated across the two care settings* *.* *’* (C14)

##### Political impact

In Japan, many stakeholders are involved in the board certification of generalist medicine practitioners, with no consensus on expertise in family medicine. Therefore, the concept of EGP may have political implications for stakeholders and for the medical profession, patients, and policymakers:

*‘EGP can be used to distinguish between those who practise it and those who do not, and this difference can help the former lead the way professionally and politically* *.* *’* (B7)

#### Negative impact

##### Disconnect among family doctors

Participants did not identify any significant negative impact; however, one doctor was concerned that EGP might divide family doctors:

*‘Considering EGP as the core expertise of family medicine might create a divide within the family doctor community, especially between those whose primary focus is EGP and those who have a special interest in other areas, such as women’s health, medical education, and research. This fragmentation would go against the professional values of medical generalism and family medicine, which inherently encourage and welcome inclusiveness and diversity* *.* *’* (C11)

### Triggers for EGP

Participants proposed target patients and situations for EGP. In addition to the original EGP targets, such as undifferentiated and complex problems, the widest range of care, and first contact care, participants mentioned *‘* *difficult patient encounters* *’* (A2), *‘* *heartsink patients* *’* (D18), *‘* *seemingly distant patients* *’* (A3), *‘* *frequent attenders of various clinics and hospitals* *’* (A3), and *‘* *those with problems that do not fit the International Classification of Primary Care code* *’* (D15).

Doctors may also need to exercise EGP with a focus on the patient’s family when patients lack the capacity to make their own decisions:

*‘If the patient is unable to make decisions about their care due to cognitive impairment, their family can be a target for EGP* *.*’ (A3)

In terms of settings, participants mentioned that:

*‘EGP was not used in situations where biomedical factors take a priority over psychosocial factors, such as in emergency rooms* *.* *’* (B8)

The following were identified as situations where EGP was required: *‘* *patients with complex needs* *’* (B9) and *‘* *consultations that may take a longer time for trainees* *’* (D17).

Participants were able to identify these situations from the medical records and information sheets used before the consultation. In addition, they categorised the level of difficulty in dealing with the stated problems.

### Enablers of EGP

Participants mentioned individual and environmental factors as enablers of EGP, and they considered these factors to be important in EGP education.

#### Individual factors

##### Ascetic practice

Some participants said that consistent ascetic practice with strict training would be essential and it is the only way to master EGP:

*‘In the past, people sat under waterfalls as part of ascetic practice to think deeply in search of enlightenment. Acquiring EGP seems somewhat similar to that in a sense* *.* *’* (D17)

They believed that EGP practice would require long-term training in the physical and mental aspects of patience:

*‘I’ve worked for a long time in a small solo practice, with many struggles along the way, and I think EGP is one of the things I’ve learnt from that experience* *.* *’* (D17)

##### Culture

Participants indicated the importance of culture in presuming the patient’s life history from their life circumstances. According to one participant, the following was a requirement for acquiring such a culture:

*‘I think this kind of thing is hard to be captured and written in textbooks. I do read articles in the relevant medical literature of course, but I also read about philosophy and Zen. I think meditation would be good, too* *.* *’* (D17)

##### Awareness of the self

Participants emphasised the importance of housekeeping and self-management:

*‘Self-management of the doctor is an important concept for EGP, I guess* *.* *’* (A2)

In addition, they reported that the ability of doctors to exercise EGP also depended on the following conditions:

*‘Both the environment in which we work and the way we manage our own health are important but whether we can acquire EGP or how well we can demonstrate it also depends on our previous personal experiences and internal aspects, I think* *.* *’* (D18)

##### Tolerance for uncertainty

Participants mentioned the importance of the ability to tolerate uncertainty:

*‘The ability to leave things as undifferentiated, the patience to deal with complex problems over time, and the ability to tolerate the unsolvable because most of these problems are unsolvable are all important, although this might sound quite simple* *.* *’* (A5)

Tackling uncertainty was identified as an issue that should be addressed in multidisciplinary teams:

*‘When you work as part of a team, you are more likely to be able to tolerate uncertainty* *… Working in a group is good for education, and I think it also helps with my self-management* *.* *’* (A1)

##### Practice of interpretive medicine

Participants reported that understanding patients’ illness experience and what they value in their life are important aspects of EGP:

*‘It’s about exploring factors that support the patient’s health and well* *being and knowing their life history and avoidance behaviours. This knowledge is stored within me and can be used one day; for example, if they need to be admitted to hospital in a few years’ time* *.* *’* (A5)

One participant focused on the patient’s life history:

*‘It’s a point where you can pull a trigger and say, “Let me find out more about this person and his or her life* *.* *”’* (A3)

#### Environmental factors

Participants thought that acquiring EGP required a long-learning period:

*‘Without enough time, it would be very difficult, if not impossible, to provide EGP* *.* *’* (D18)

Task-sharing was seen as key to ensuring that enough time would be available:

*‘Doctors need the support of other healthcare professionals so that they can share tasks. It may be the doctor who ultimately brings everything together, but I think it’s a good idea to have someone who can be trusted with certain elements of those tasks* *.* *’* (D18)

They also suggested that working in a multidisciplinary team to address the care of patients together would help promote EGP:

*‘Working in a supportive environment, like in a multidisciplinary team of nurses and administrative staff and so on, would help and enhance EGP* *.* *’* (A3)

The need for a greater change in the medical system was also mentioned:

*‘Seeing patients with complex problems is not directly linked to reimbursement, is it? This is the current payment system in Japan* *.* *’* (D18)

### Possible strategies for education

In addition to the enablers mentioned above, participants suggested teaching strategies for EGP education. The suggested strategies were categorised into ‘on-the-job training’ and ‘developing a framework’.

#### On-the-job training

The usefulness of ‘reflection’ was repeatedly mentioned in different groups:

*‘I think it’s really reflective practice that is key for learners* *.* *’* (A1)

Some participants proposed that learners watch video-recordings of their consultations with instructors to help them reflect on their practice. One participant also stated that it was difficult to teach EGP, and that the only way to do this would be to provide opportunities for reflection. One participant used the phrase ‘time out’ to describe the usefulness of creating opportunities to pause and reflect on one’s practice:

*‘It’s like a time out. After the consultation, we can step back and reflect with the trainee on what was happening. We can check together and visualise their progress* *.* *’* (A3)

Participants identified two methods of learning EGP: *‘* *practising with the support of a supervisor and learning it in the process* *’* and *‘* *learning from watching a supervisor* *’* (as opposed to being taught verbally). Both meant that, currently, learners only observed EGP, and that instructors did not intend to teach EGP:

*‘Rather than teaching* [trainees]*, we want them to experience it first-hand. If you do not experience it, you will not have a real sense of it; thus, we experience and think about it together* *.* *’* (C11)

Presenting difficult cases in case conferences was also considered useful for the dissemination of tacit knowledge and solutions:

*‘I think the only way to learn about difficult cases is to learn from actual cases. In our area, we learn from listening to people who are actually involved in such cases in community care meetings* *.* *’* (C14)

#### Developing a framework

Some participants thought that clearer conceptualisation and verbalisation of EGP is an important step in teaching:

*‘The concept of EGP is too vague for me to be able to share with others or teach to trainees* *.* *’* (B8)

Learning through a clearly developed framework and clinical scenarios may also help overcome education difficulties attributable to the ambiguity of EGP:

*‘Something like checklists or scoring would be good for beginners, I think.* […] *When trainees are asked to provide EGP in a real clinical setting, it would be difficult to do so without first practising it in a scenario* *.* *’* (B8)

Meanwhile, they also acknowledged that developing such a framework could interfere with the understanding of EGP:

*‘If we tell trainees too much about what to do, we risk making EGP mechanical and something they have to do and not being able to convey the true value and benefits of EGP. We might actually end up losing its intrinsic appeal* *.* *’* (B8)

**Figure 1.**
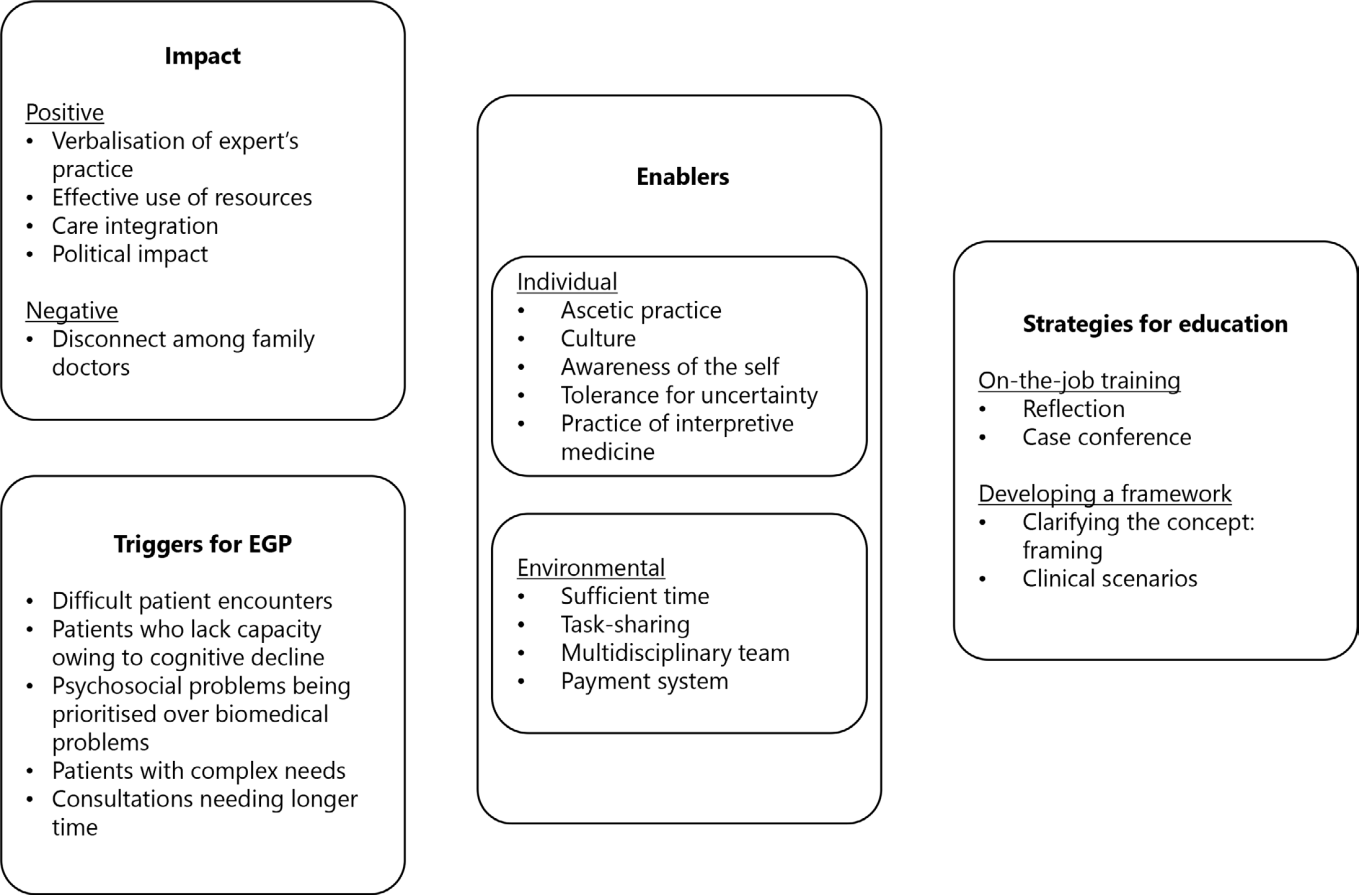
Japanese family doctors’ perspectives on expert generalist practice (EGP)

Participants’ views on EGP have been summarised in Figure 1.

## Discussion

### Summary

This study explored Japanese family doctor educators’ views on EGP. The participants considered difficult and complex patient care as an important candidate area for EGP. This concept enabled them to demonstrate the value of their practice in dealing with patients with complex health needs more clearly, which was often overlooked in Japanese health care. This point seemed to encourage several participants because primary care is not considered to be of paramount importance in Japan. Some participants mentioned that families of patients who lack the capacity to make medical decisions owing to cognitive decline should also be candidate recipients of EGP. This may reflect the nature of the work of family doctors in Japan, who are tasked with dealing with a super-ageing population. Further, it highlights the importance of family support in this context.

Participants offered a wide range of suggestions for educational strategies. Some called for a clearer explanation of the concept of EGP or for the development of a framework to make it simpler and easier to understand. Some participants felt that EGP mapped to the professional values, but its concept was not clear and was difficult to teach to trainees. Therefore, they seemed to pursue a systematic approach to teaching EGP, in line with the current framework of medical education, rather than accepting EGP as it is presented currently. This attempt to simplify EGP was in contrast to alternative educational methods to master EGP, such as reflection, ascetic practice, and awareness of the self. These views may indicate an acceptance of the ambiguity of the concept, as these methods, particularly reflection, were seen as useful for dealing with complex and ambiguous problems. It was also suggested that a self-assessment tool about EGP may be helpful to learners.

### Strengths and limitations

Although this study provided valuable insights into the concept of EGP from the perspective of family doctors in Japan, it has several limitations. First, the study mainly included directors or associate directors of family medicine training programmes because the authors wanted to collect the views of family doctor educators, which resulted in missing the views of junior doctors and other professionals.

Second, patients’ views were not explored. Indeed, while deciding whether this concept should be implemented in health care and medical education, it is important to understand how this concept impacts patients’ experience of care. However, it was not possible to include patients in the present study owing to limited time and resources. Future studies could focus on this issue.

Finally, the interviews were conducted in Japanese, and the results were reported in English. Some important information may have been lost during translation. Recognising the cultural issues involved in this topic, the research team included a British GP who is familiar with the Japanese culture, and a Japanese GP who practises in the UK, speaks Japanese as a native language, and is familiar with the Japanese culture. This allowed the results from different cultural perspectives to be discussed. It also ensured that all the translated quotes conveyed the meaning and nuances of the original text adequately.

### Comparison with existing literature

The primary components of EGP are ‘person-centred decisionmaking’ and ‘interpretive medical practice’,^6^ and, as confirmed in the present study, family doctor educators in Japan also viewed these elements as critical to their practice, acknowledging that this is the core expertise of generalist care.^8^ In addition, Japanese family doctor educators cited care integration and effective use of healthcare resources as positive impacts of EGP, consistent with earlier findings.^7^ Reeve *et al* ^7^ identified *‘* *lack of a consistent understanding of distinct expertise* *’*, *‘* *competing priority inhibiting EGP* *’*, *‘* *lack of consistent development of skills in interpretive practice* *’*, and *‘* *lack of resources for manageable monitoring building* *’*. Similar to those identified by Reeve *et al*,^7^ the present study identified ‘difficulty in accessing those who have mastered EGP’ and ‘ambiguity in the concept of EGP’ as the primary barriers to EGP. To overcome these barriers, Reeve *et al* ^7^ recommended *‘* *articulating the concepts* *’* *,* *‘* *revisiting risk stratification* *’* *,* *‘* *extending training and continuous professional development* *’* *,* and *‘* *basing on evidence for generalist practice* *’*. While some remarks were made regarding the usefulness of developing an educational framework for EGP in the present study, participants mentioned that the essential characteristics of EGP may unintentionally be lost through these processes. The importance of reflecting on one’s own practice to learn about EGP was also noted in the present study. The School for Advancing Generalist Expertise (SAGE) model, a practice model of EGP, also includes reflection with colleagues,^20^ which is in keeping with the present findings. In addition, Japanese family doctor educators identified the creation of an environment conducive to EGP, such as the promotion of self-management and multidisciplinary collaboration, as a necessary element for the development of EGP, which may be useful in other countries as well.

### Implications for research and practice

The study suggested that the concept of EGP may be useful in clinical practice in Japan. It is particularly significant that the concept of EGP confirms the value of ‘complex patient care’. The care of patients with complex needs, which is especially prevalent in ageing societies, is one of the key roles of family doctors, and it is one of the competencies that characterises their expertise. The Japanese family doctors participating in the study expressed that competence in and value of caring for patients with difficult and complex issues were not well respected. They also noted inadequate allocation of healthcare resources. Evidently, with the spread of the concept of EGP, the value of caring for patients with complex health issues would receive more recognition. This, in turn, would lead family doctors to consider complex patient care more rewarding, enabling them to explain the differences between primary care doctors and family doctors with formal training and practice in the community. Thus, EGP has the potential to shed light on the complex patient care provided by family doctors — which has traditionally been under-recognised — and, as a result, encourage family doctors’ practice. However, it is still unclear how EGP affects the process and outcomes of patient care and patients’ illness experience. These aspects need to be clarified in future studies.

Currently, the concept of EGP is considered abstract. Therefore, a clearer, more specific description of EGP would facilitate its integration into current medical education. Some participants suggested developing a straightforward framework to support this, or holding educational workshops. Others suggested that ascetic practice and awareness of the self, which are currently not common in medical education, should be included. These participants seemed to view EGP as an art of medicine based on humanism rather than a strategy for delivering more effective primary care. Therefore, the method of creating the so-called framework proposed by some participants may not be sufficient to educate practitioners on EGP. The methods of teaching or mastering EGP need to be clarified in future research.

The study revealed Japanese family doctor educators’ perceptions of EGP. The concept of EGP was perceived to be important and relevant, and participants were able to relate it to their practice. A clearer framework and explanation of the concept, as well as the use of non-traditional teaching methods, such as ascetic practice and awareness of the self, were proposed as possible educational strategies.
